# Quantifying the risk of respiratory infection in healthcare workers performing high-risk procedures

**DOI:** 10.1017/S095026881300304X

**Published:** 2013-12-05

**Authors:** C. R. MACINTYRE, H. SEALE, P. YANG, Y. ZHANG, W. SHI, A. ALMATROUDI, A. MOA, X. WANG, X. LI, X. PANG, Q. WANG

**Affiliations:** 1 School of Public Health and Community Medicine, UNSW Medicine, University of New South Wales, Australia; 2 Beijing Centre for Disease Prevention and Control, Beijing, China

**Keywords:** Aerosol-generating procedures, healthcare workers, high-risk procedures, respiratory infections

## Abstract

This study determined the risk of respiratory infection associated with high-risk procedures (HRPs) performed by healthcare workers (HCWs) in high-risk settings. We prospectively studied 481 hospital HCWs in China, documented risk factors for infection, including performing HRPs, measured new infections, and analysed whether HRPs predicted infection. Infection outcomes were clinical respiratory infection (CRI), laboratory-confirmed viral or bacterial infection, and an influenza infection. About 12% (56/481) of the study participants performed at least one HRP, the most common being airway suctioning (7·7%, 37/481). HCWs who performed a HRP were at significantly higher risk of developing CRI and laboratory-confirmed infection [adjusted relative risk 2·9, 95% confidence interval (CI) 1·42–5·87 and 2·9, 95% CI 1·37–6·22, respectively]. Performing a HRP resulted in a threefold increase in the risk of respiratory infections. This is the first time the risk has been prospectively quantified in HCWs, providing data to inform occupational health and safety policies.

## INTRODUCTION

Healthcare workers (HCWs) are at increased risk of healthcare-associated infections due to the front-line nature of their work. Transmission of highly infectious diseases from infected patient to other patients and HCWs occurs constantly in hospitals and healthcare centres and has been well documented [1–3]. Although HCWs are aware of infection control measures, low levels of compliance with standard precautions by this group are frequent [4, 5]. HCWs are less willing to adhere to infection-control practices when they work for extended hours [6], with probable reasons for low compliance being insufficient time, scarcity of equipment, lack of knowledge and low perception of risk [5].

The three principal routes of transmission of respiratory pathogens are contact transmission (direct and indirect), droplet transmission, and airborne transmission. For any pathogen, more than one transmission route may occur, but many pathogens are known to be transmitted by one predominant mode. In droplet transmission, pathogens or droplets which are larger than 5 *μ*m, such as influenza virus and *Bordetella pertussis* are transmitted from an infected patient to HCWs through breathing, talking, coughing, sneezing, as well as through performing high-risk procedures (HRPs) [2, 7, 8]. However, influenza virus has also been documented to be transmitted by the airborne route, which results in infectious particles being present in the air for longer periods of time [9–12].

Respiratory infectious diseases, even those with limited airborne transmission, are more likely to be transmitted from patients to HCWs during HRPs such as suctioning and intubation which generate respiratory aerosols [13]. Many studies suggest that both invasive and non-invasive procedures are likely to increase the probability of HCWs being infected [13, 14]. Some studies have reported that non-invasive positive pressure ventilation (NPPV) can be a risk of severe acute respiratory syndrome (SARS) transmission to HCWs [15–17]. Cardiopulmonary resuscitation, manual ventilation, bronchoscopy and suctioning have also been documented to increase the risk of HCWs being infected with SARS and tuberculosis (TB) [18–21], while tracheal intubation has been significantly associated with risk of SARS transmission to HCWs [17]. While it has been well documented that TB and SARS can be transmitted to HCWs during aerosol-generating procedures, there are some data suggesting H1N1 can transmit via such procedures [13]. Seasonal influenza also causes outbreaks in healthcare settings [22]. HCWs are one of the most vulnerable groups likely to be infected with influenza infection in acute-care facilities due to the high exposure rates in such settings [23]. An attack rate of nosocomial influenza could reach 11–59% in HCWs in a healthcare environment [24]. As such, HCWs are a priority group for preventive strategies such as influenza vaccination [25, 26].

Although various guidelines and policies for infection control measures are implemented in healthcare settings worldwide, the risk of transmission of infectious diseases while performing HRPs has not been well quantified. This study aims to describe the range of exposure to HRPs in HCWs and to quantify the risk of respiratory infections occurring in HCWs who perform HRPs.

## METHODS

We prospectively studied 481 hospital HCWs from wards including emergency and respiratory wards from nine hospitals in Beijing, China over a 5-week period from 1 December 2008 to 15 January 2009. These 481 subjects were a control group in a larger study [27].

The hospital wards were selected as high-risk settings in which repeated and multiple exposures to respiratory infections are expected. Participants were hospital HCWs aged ⩾18 years and who were provided with written information about the study. Staff who agreed to participate provided informed consent and a copy of the information sheet with the participants' initials was retained as documentation [27]. The study protocol was approved by the Institutional Review Board and Human Research Ethics Committee of the Beijing Ministry for Health.

Participants were asked to record on a daily basis whether they had performed one of the following: provision of nebulizer medications, suctioning, intubation, aerosol-generating procedures and chest physiotherapy. The following information was also collected: number of hours worked, estimated number of daily contacts with patients, number of daily contacts with influenza-like illness (ILI) patients, and hand-washing adherence. The use of personal protective equipment such as gloves, gowns, eye shields, foot/hair covers was documented by study participants in a daily self-report diary, and details of clinical and demographics were also recorded. All study participants were followed up for a period of 31 days and monitored daily for the onset of respiratory symptoms.

**Fig. 1.**
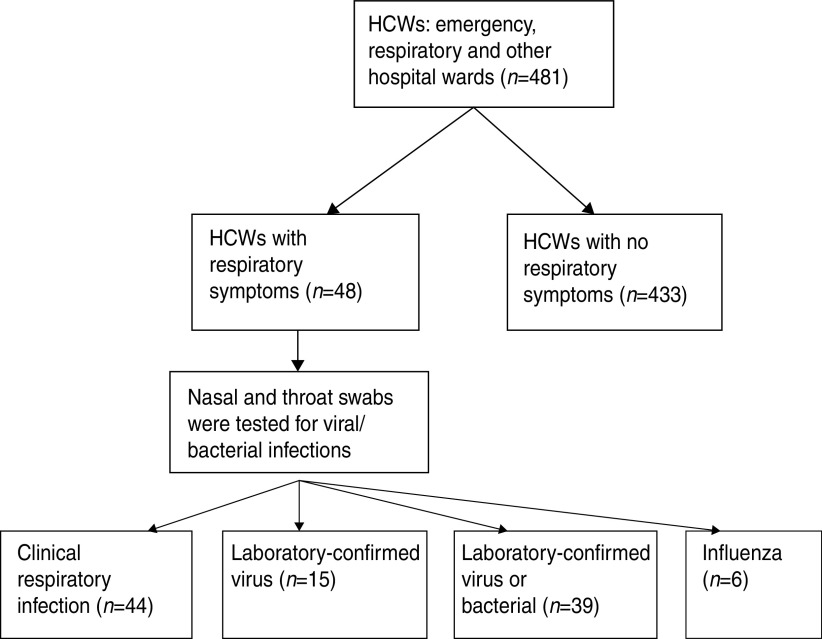
Flow diagram for the study recruitment. HCWs, Healthcare workers.

If any symptom developed, combined nasal and throat swabs (double rayon-tipped, plastic-shafted swab) were taken and tested for respiratory viral or bacterial infection (Fig. 1). The nose and throat swabs were tested at the Laboratories of the Beijing Centres for Disease Control and Prevention. Viral DNA⁄RNA was extracted from 300*μ*l of each respiratory specimen using the Viral Gene-spin^™^ kit (iNtRON Biotechnology Inc., Korea) according to the manufacturer's instructions [27]. We tested nose and throat swabs for the following: adenoviruses, human metapneumovirus (HMP), coronaviruses 229E/NL63 and OC43/HKU1, parainfluenza viruses 1, 2 and 3, influenza viruses A and B, respiratory syncytial virus (RSV) A and B, and rhinovirus A/B by nucleic acid testing using a commercial multiplex polymerase chain reaction (PCR), with the Seeplex® RV12 Detection kit (Seegen Inc., Korea). Details of laboratory methods have been described in a previous publication [27]. We also tested for bacterial colonization. A multiplex PCR (Seegen Inc.) was used to detect *Streptococcus pneumoniae, Mycoplasma pneumoniae, B. pertussis, Legionella* spp, *Chlamydophilia* and *Haemophilus influenza* type B. After preheating at 95 °C for 15 min, 40 amplification cycles were performed under the following conditions in a thermal cycler (GeneAmp PCR system 9700, Applied Biosystems, USA): 94°C for 30 s, 60°C for 1·5 min, and 72°C for 1·5 min. Amplification was completed at the final extension step at 72°C for 10 min. The multiplex PCR products were visualized by electrophoresis on an ethidium bromide-stained 2% agarose gel.

The controls represent HCWs in their usual working conditions, without any interventions. This study is a *post-hoc* analysis of data collected during the primary trial on HRPs in the control arm. The prospective data collection and measurement of clinical endpoints in a group of HCWs working under usual conditions afforded the opportunity to measure the association of incident infection with HRPs.

The primary outcomes of the study were: clinical respiratory infection (CRI) – presence of two or more respiratory symptoms or one respiratory symptom (e.g. cough, runny nose, shortness of breath, sore throat) and one systemic symptom (e.g. fever, lethargy, chills); laboratory-confirmed viral infection (influenza A and B, parainfluenza, RSV, coronavirus, HMP virus, adenovirus, rhinovirus); laboratory-confirmed viral or bacterial infection (and of the above viruses or a bacterial infection – pertussis, Hib, pneumococcus, *Mycoplasma, Legionella*); and influenza A or B (categorized as ‘influenza’ if either strain were present). The outcomes were tested against predictor variables such as age, education, category of HCW, influenza vaccination uptake, and performance of hand washing and HRPs. The total number of HRPs performed over the study period was calculated. A binary variable defining whether or not HCWs performed any HRPs during the study period was created and analysed with other predictor variables against incident infection during the study period. Poisson regression was used for the analysis of the outcomes, using Egret software (Cytel, USA). A *P* value of ⩽0·05 was considered significant in the analysis.

## RESULTS

A total of 481 HCWs were recruited into the study. Demographic characteristics of study participants are described in Table 1. Of these, 369 (76·7%) were females; and 52% (252/481) of the participants were aged ⩾35 years. The breakdown of participants by area was: respiratory ward 75 (16%); emergency department 72 (15%); respiratory clinic 16 (3·3%); paediatric department 15 (3·1%); infection fever clinic 6 (1·2%); and other wards 297 (62%). Of the 481 HCWs, 236 (49%) were doctors and 245 (51%) were nurses and others. During the study period, the uptake of influenza vaccine in HCWs was low in both 2007 and 2008 (19·3% and 18·1%, respectively).

**Table 1.** Demographic characteristics of healthcare workers in the study

	Number (%)
Sex	
Male	112 (23·3)
Female	369 (76·7)
Age group	
<35 years	229 (47·6)
⩾35 years	252 (52·4)
Ward/department	
Emergency medical	72 (15)
Infection fever clinic	6 (1·2)
Respiratory clinic	16 (3·3)
Respiratory ward	75 (15·6)
Paediatric medical department	15 (3·1)
Other	297 (61·7)
Staff	
Doctor	236 (49·1)
Nurse and other	245 (50·9)
Hgh-risk procedure performed	
Yes	56 (11·6)
No	425 (88·4)

**Fig. 2.**
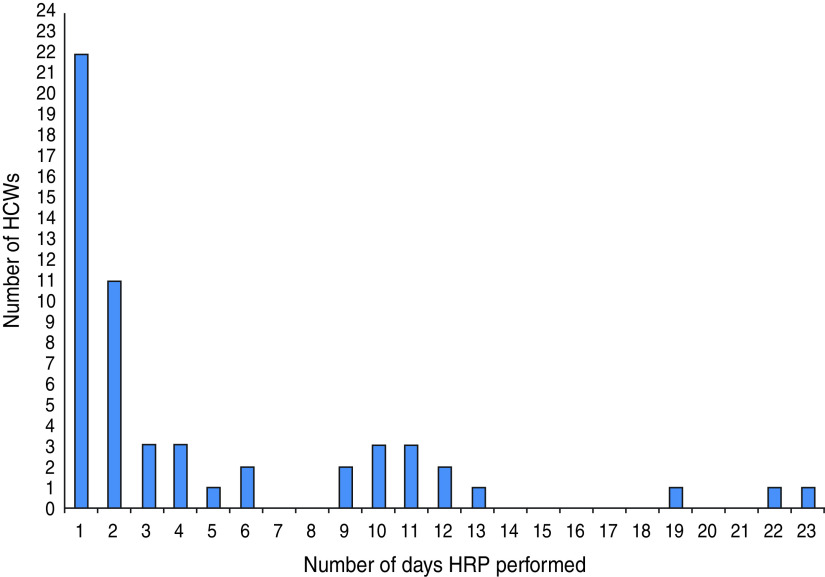
High-risk procedures (HRPs) performed by healthcare workers (HCWs).

Fifty-six (11·6%) out of 481 HCWs performed at least one HRP during the study, with the most common activity being airway suctioning (66%, 37/56). Figure 2 shows the number of days on which a HCW reported performing a HRP. Thirty-four (61%) out of 56 HCWs, reported performing a HRP more than once during the study period. The aggregated number of days a HRP was performed was 264. In addition, HCWs on the respiratory ward (33%) were more likely to perform HRPs than those in the emergency department (16·7%). Nurses and other HCWs (16%, 39/245) were significantly more likely than doctors (7%, 17/236) to perform HRPs (*P* < 0·01).

The weekly incidence of CRI was 18/1000 HCWs, for viral or bacterial infection, 16/1000; for any viral infection, 6/1000; and for influenza, 2·5/1000. HCWs who performed HRPs had a significantly higher risk of CRI [relative risk (RR) 2·5, 95% (CI) 1·3–6·5, *P* < 0·01) and laboratory-confirmed viral or bacterial infection (RR 2·6, 95% CI 1·4–5, *P* < 0·01) than those who did not perform HRPs (Table 2). By Poisson regression analysis, adjusting for other variables, only HRPs determined the risk of an infection outcome, as shown in Tables 3–5. The relative risk for CRI in HCWs who performed a HRP was 2·9 (95% CI 1·42–5·87, *P* < 0·01) (Table 3). The RR for a laboratory-confirmed pathogen (viral or bacterial) in symptomatic HCWs was 2·9 (95% CI 1·37–6·22, *P* = 0·01), in those who performed a HRP (Table 4). For the outcome of any respiratory viral pathogen, the RR was 3·3 (95% CI 1·01–11·02, *P* = 0·05) (Table 5). Hand washing, influenza vaccination and use of surgical or cloth face masks did not affect the risk of infection outcomes.

**Table 2.** Respiratory outcomes in healthcare workers who did and did not perform HRPs*, univariate analysis

	HRP performed*	HRP not performed	RR	95% CI	*P* value
Clinical respiratory infection	11/56 (20%)	33/425 (7·7%)	2·5	1·3–6·5	<0·01
Laboratory-confirmed virus†	4/56 (7%)	11/425 (2·6%)	2·8	0·9–8·7	0·07
Laboratory-confirmed virus or bacteria‡	10/56 (18%)	29/425 (7%)	2·6	1·4–5	<0·01
Influenza	1/56 (1·7%)	5/425 (1·1%)	1·5	0·2–13	n.s.

RR, Relative risk; CI, confidence interval; n.s., not significant.

* HRP, High-risk procedure, defined as healthcare workers who performed high-risk procedures at least once during the study period.

† Viruses detected were: human coronavirus 229E/NL63 (*n* = 1), influenza B (*n* = 2), influenza A (*n* = 4), rhinoviruses (*n* = 3), respiratory syncytial viruses (*n* = 5).

‡ Bacteria detected were *Streptococcus pneumonia* (*n* = 30) and *Haemophilus infleunzae* (*n* = 24).

**Table 3.** Risks of clinical respiratory infection in healthcare workers, Poisson regression analysis

	RR	95% CI	*P* value
Age (<35 years)	0·8	0·44–1·52	0·52
Education (Undergraduate, Master, PhD)	1·0	0·42–2·25	0·94
Doctor	1·3	0·53–3·08	0·59
Influenza vaccine	1·0	0·48–2·28	0·92
Hand washing	0·7	0·31–1·51	0·35
HRP* performed	2·9	1·42–5·87	**<0·01**

RR, Rate ratio; CI, confidence interval.

* HRP, High-risk procedure, defined as healthcare workers who performed high-risk procedures at least once during the study period.

**Table 4.** Risks of laboratory-confirmed virus or bacteria in healthcare workers, Poisson regression analysis

	RR	95% CI	*P* value
Age (<35 years)	1·0	0·5–1·9	0·94
Education (Undergraduate, Master, PhD)	0·6	0·26–1·52	0·3
Doctor	2·3	0·86–6·11	0·1
Influenza vaccine	1·3	0·56–2·8	0·58
Hand washing	0·7	0·3–1·63	0·41
HRP* performed	2·9	1·37–6·22	**0·01**

RR, Rate ratio; CI, confidence interval.

* HRP, High-risk procedure, defined as healthcare workers who performed high-risk procedures at least once during the study period.

**Table 5.** Risks of laboratory-confirmed virus in healthcare workers, Poisson regression analysis

	RR	95% CI	*P* value
Age (<35 years)	1·3	0·46–3·82	0·59
Education (Undergraduate, Master, PhD)	1·3	0·28–5·77	0·75
Doctor	1·7	0·36–8·29	0·5
Influenza vaccine	1·3	0·35–4·71	0·7
Hand washing	0·6	0·15–2·01	0·36
HRP* performed	3·3	1·01–11·02	**0·05**

RR, Rate ratio; CI, confidence interval.

* HRP, High-risk procedure, defined as healthcare workers who performed high-risk procedures at least once during the study period.

We also tested for other variables, e.g. number of hours worked, number of patients the HCW was in contact with during the study period, and number of contacts with patients with ILI; however, none of these had a significant association with infection outcomes. There were no significant association between laboratory-confirmed influenza and HRPs (data not shown); but the numbers of influenza-positive cases were low (six cases) in the study.

## DISCUSSION

We examined the association between HRP and the risk of respiratory infection in HCWs. Our findings demonstrated that HCWs who perform a HRP have a greater risk of respiratory infections than those who did not perform a HRP. This is consistent with observational findings of other studies [15, 16, 18, 21, 28]; however, we have been able to quantify the magnitude of this risk in our study as a threefold increase in risk.

Many factors influence the nosocomial spread of infectious diseases, and HCWs are the initial point of contact with patients in both acute and long-term healthcare settings. Our findings suggest that targeted interventions and policies are warranted to offer greater protection to HCWs who perform HRPs. This has occupational health and safety implications for HCWs routinely engaged in HRPs. More than 10% of HCWs performed a HRP during a 1-month period, and the majority of those performed more than one HRP. This suggests that interventions to reduce transmission of respiratory infections may be more efficient if targeted to HCWs performing HRPs. There may be certain settings such as emergency wards, intensive-care units and respiratory wards where HRPs are more commonly performed, making these important targets for interventions.

There are some limitations to our study. Our study was conducted in China, so the results, particularly around frequency of performing HRPs, may not be generalizable to different HCW populations in other contexts. There are variations in infection control practices from hospital to hospital, even within China. However, the quantification of risk for HCWs who perform HRPs has implications for HCWs everywhere. The fact that this was a control group in a larger trial is a strength, rather than a limitation, in that this group had rigorous follow-up and documentation of incident infection as well as risk factors (including HRPs). This provides more robust data than, for example, an observational study such as a case-control study, because it was prospective and measured infection in a group that went about usual practice. To date, there is much policy debate and direction about HRPs, but no data whatsoever to inform the actual risk associated with HRPs. Despite the limitations of the analysis, we believe that the data we present in this paper are a useful addition to current knowledge.

HCWs are at higher risks of contracting respiratory infections, and are subject to generic guidelines around infection control. These include hand hygiene before and after the patient care; wearing of personal protective equipment such as gowns, goggles, gloves, N95 respirators or surgical masks; presence of minimum number of HCWs when performing a procedure in a single room; and in addition, it is recommended that such procedures should be performed in a sterilized room [13, 29, 30]. We have shown in two large randomized controlled trials that the risk of respiratory infection in HCWs can be reduced with the use of N95 respirators [27, 31]. We also show that in high-risk wards, targeted use in situations of self-identified risk, such as when performing HRPs or barrier nursing a patient, is less effective than continuous use of a respirator in that ward while on shift [31]. This suggests that HCWs are unable to identify all situations of risk when left to decide whether or not they should wear a respirator.

This is the first time the risk of HCWs performing HRPs has been prospectively quantified, and this finding has important occupational health and safety implications for HCWs, particularly in settings such as emergency and respiratory wards where HRPs are frequently performed. The traditional approach to hospital infection control has not consistently categorized staff in terms of whether they perform HRPs in order to apply guidelines. We found that the majority (89%) of HCWs do not perform HRPs. This proportion may vary in different country, hospital and ward settings; our study suggests that categorizing HCWs by whether or not they perform HRPs in their work may serve as a useful classification in order to tailor guidelines appropriately or increase the attention to adherence with existing guidelines. The minority of HCWs performing HRPs should receive optimal respiratory protection, and high-risk wards should have guidelines in place to minimize the risk to HCWs.
